# Simultaneous detection of three pome fruit tree viruses by one-step multiplex quantitative RT-PCR

**DOI:** 10.1371/journal.pone.0180877

**Published:** 2017-07-27

**Authors:** Ioanna Malandraki, Despoina Beris, Ioannis Isaioglou, Antonio Olmos, Christina Varveri, Nikon Vassilakos

**Affiliations:** 1 Benaki Phytopathological Institute, Department of Phytopathology, Laboratory of Virology, Athens, Greece; 2 Plant Protection and Biotechnology Centre, Instituto Valenciano de Investigaciones Agrarias (IVIA), Moncada, Valencia, Spain; National University of Singapore, SINGAPORE

## Abstract

A one-step multiplex real-time reverse transcription polymerase chain reaction (RT-qPCR) based on TaqMan probes was developed for the simultaneous detection of *Apple mosaic virus* (ApMV), *Apple stem pitting virus* (ASPV) and *Apple stem grooving virus* (ASGV) in total RNA of pome trees extracted with a CTAB method. The sensitivity of the method was established using in vitro synthesized viral transcripts serially diluted in RNA from healthy, virus-tested (negative) pome trees. The three viruses were simultaneously detected up to a 10^−4^ dilution of total RNA from a naturally triple-infected apple tree prepared in total RNA of healthy apple tissue. The newly developed RT-qPCR assay was at least one hundred times more sensitive than conventional single RT-PCRs. The assay was validated with 36 field samples for which nine triple and 11 double infections were detected. All viruses were detected simultaneously in composite samples at least up to the ratio of 1:150 triple-infected to healthy pear tissue, suggesting the assay has the capacity to examine rapidly a large number of samples in pome tree certification programs and surveys for virus presence.

## Introduction

Pome fruit cultivation constitutes one of the most economically important tree cultivations in the world. *Apple mosaic virus* (ApMV, genus *Ilarvirus*), *Apple stem pitting virus* (ASPV, genus *Foveavirus*) and *Apple stem grooving virus* (ASGV, genus *Capillovirus*) are distributed worldwide and constitute important virus pathogens for both apple (*Malus × domestica* Borkh.) and pear (*Pyrus* spp.) crops [1–4]. Their main means of transmission is through infected propagation plant material. Single infections with ASPV and ASGV are symptomless in apple but can cause diverse symptoms in pear like vein yellowing or red mottling, and pear black necrotic leaf spot, respectively [2, 3, 5–8]. In addition, mixed infections of the three viruses are very common and can cause complex disease symptoms in both apple and pear trees, resulting in significant reduction in quality and yield [1, 2, 9–11]. The genetic analyses of ApMV, ASGV and ASPV showed extensive genomic diversity [3, 12–18].

The main control strategies as with any other plant viral disease rely on early detection, eradication and use of genetically resistant or tolerant cultivars. The use of certified planting material and germplasm for these three viruses along with other pathogens [4], is a prerequisite for the global trade of pome trees and depends on the application of reliable, sensitive and fast detection methods. Thus far, several molecular, reverse transcription polymerase chain reaction (RT-PCR) assays have been developed and used for single or simultaneous detection of pome tree viruses [19–26].

The development of real-time quantitative PCR (qPCR) based methods led to superior sensitivity, speed, reproducibility and limited risk of contaminations compared to conventional RT-PCR. These characteristics often make it the method of choice in routine diagnostics. The possibility of multiplexing, allowing simultaneous detection of different targets in one sample, makes RT-qPCR even more appealing to diagnosticians and epidemiologists [27, 28]. Singleplex reverse transcription real-time quantitative PCRs (RT-qPCR) using molecular beacons, TaqMan probes or SYBR-Green chemistries, have been developed for ASPV, ASGV and ApMV [29–33], however, no multiplex RT-qPCR for the simultaneous detection of the three viruses in pome trees has been reported.

The objective of this study was to develop a fast, sensitive and reliable assay for the simultaneous identification of ASPV, ASGV and ApMV in pome fruit trees.

## Materials and methods

### Virus isolates and plant material

Virus reference isolates were provided by the Centre Technique Interprofessionnel des Fruits et Légumes (CTIFL, France). An apple tree (B71) naturally triple-infected with ApMV, ASPV and ASGV was used along with reference isolates ApMV7, ASPV10 and ASGV13 for the assay development (S1 Table). The assay specificity was assessed using reference isolates as well as isolates of unrelated pathogens such as *Apple chlorotic leaf spot virus* (ACLSV), *Apple scar skin viroid* (ASSVd) and *Pear blister canker viroid* (PBCVd) from the collections of Benaki Phytopathological Institute (BPI, Greece) and CTIFL. The assay specificity was evaluated with healthy pear, apple and quince (*Cydonia oblonga* Mill.) tissues. Validation and comparative analysis was performed on 36 plant samples derived from commercial orchards located in different parts of Greece.

### Nucleic acids extraction

Total RNA was extracted from pear and apple leaves. Due to possible uneven virus distribution, leaf discs were collected from at least two different younger branches located at the upper one third of the tree. Leaf discs were mixed together and homogenized using liquid nitrogen.

Considering the presence of compounds that bind or co-precipitate with the RNA of pome fruit trees (such as polysaccharides, polyphenolics etc.) different extraction protocols and commercial kits were examined in pear tissues; TRI Reagent Solution (Ambion) was applied with the modification for polysaccharide contamination noted in the product manual, and a pre-treatment of crude plant extract in a dilution buffer described by Rowhani and co-workers [34]. Purelink RNA Mini Kit (Ambion) was used following the manufacturer’s instructions and two additional modifications of sample preparation: one described by MacKenzie and co-workers [35] and one described by López-Fabuel and co-workers [36]. A CTAB protocol for woody plants, was applied as described by Gambino and co-workers [37].

In order to simulate composite sample examination, leaf samples from a naturally triple infected apple tree (B71) were mixed with virus negative tested pear leaf tissue in ratios of 1:25, 1:50, 1:100 and 1:150 before the extract preparation.

Quality (OD 260/280) and concentration of nucleic acids were determined using a NanoPhotometer^™^ P-Class P330 (IMPLEN). All RNA extracts were stored as 150 ng/*μ*L aliquots at −80°C until use.

### Conventional RT-PCR

The newly developed multiplex assay was compared to conventional RT-PCR assays. To obtain direct comparison between methods in copy number detection, primers for conventional PCR were selected to amplify products containing the same target sequence as the respective MGB probes (Table 1).

**Table 1:** Primers used for conventional assays and transcript preparation.

Target	Primer	Sequence (5΄- 3΄)	Amplicon size (bp)	Reference
**ASPV**	qASP-F	TGCCTTTTACGCAAAGCATGT	385	This study
	ASPV-R	TTGGGATCAACTTTACTAAAAAGCATAA		Menzel et al. [24]
**ASGV**	ASGV-F	CCCGCTGTTGGATTTGATACACCTC	491	James [38]
	ASGV-R	CACGACTCCTA ACCCTCCAGTTCC		Massart et al. [23]
**ApMV**	ApMV-F	GACTTTGCCGATGTCTTCCG	325	This study
	ApMV-R	GTGGTAACTCACTCGTTATCACGTAC		

Sequences of forward (F) and reverse (R) primers used for the detection of *Apple stem pitting virus* (ASPV), *Apple stem grooving virus* (ASGV) and *Apple mosaic virus* (ApMV) by conventional RT-PCR and the preparation of RNA transcripts.

Reverse transcription of RNA was performed with MMLV Reverse Transcriptase (Invitrogen) following manufacturer’s instructions, at denaturation temperature 65°C for ASPV and ApMV, and 70°C for ASGV. Typically, PCR amplification consisted of an initial cycle of 95°C for 5 min, followed by 40 cycles of 94°C for 30 s, appropriate for each pair of primers annealing temperature for 30 s (55°C for ASPV, 56°C for ApMV, 59°C for ASGV), 72°C for 60 sec and a final extension step of 72°C for 7 min. All PCR assays were performed in 25 *μ*L reactions with 2 *μ*L of 5-fold diluted cDNA as template on a Veriti Thermal Cycler (Applied Biosystems) with *Taq* DNA Polymerase (Invitrogen).

### Preparation of standards

RNA transcripts were synthesized *in vitro* and used as standards in the development of the RT-qPCR assays essentially as described previously [39]; basically, PCR products containing the qPCR target sequences were produced by conventional RT-PCR as described above, and cloned into pCRII-TOPO TA vector (Invitrogen). After sequencing verification of the obtained clones, RNA transcripts were synthesized using MEGAscript T7 (Ambion) and SP6 (Roche) Transcription Kits, according to the manufacturer's instructions. Removal of the DNA template from the *in vitro* transcription reaction was done with TURBO DNase (Ambion) as described in manufacturer’s instructions. Standards were quantified using NanoPhotometerTM P-Class P330 (IMPLEN) and the number of standard copies per nanogram was calculated using the following equation: copies per nanogram = (*N*_A_ x A) / (*n* x mw), where *N*_A_ the Avogadro constant (6.02 x 10^23^ molecules per mole), A the amount of the standard in g, *n* the length of the standard in nucleotides, and mw the molecular weight per nucleotide (considering average molecular masses of 340 Da for one nucleotide of single-stranded RNA). All primers used for standards preparation are described in Table 1.

### Primers and probes

TaqMan MGB (Minor Groove Binder) probes and primers for RT-qPCR were designed using Primer Express Software v3.0.1 (Applied Biosystems). The most conserved region of the capsid protein (CP) of each virus was identified after alignment of all full-genome and CP sequences retrieved from NCBI GenBank database. Specificity of the designed primers and probes was tested using Basic Local Alignment Search Tool (BLAST). All primers and probes are listed in Table 2. ApMV probe was VIC labelled at the 5΄end, ASPV probe was NED labelled and ASGV probe was 6-FAM labelled (Applied Biosystems).

**Table 2:** Primers and probes of the RT-qPCR assay.

Target	Primer/probe	Sequence (5΄-3΄)	Reporter dye	Amplicon size (bp)	Reference
**ASPV**	qASP-F	TGCCTTTTACGCAAAGCATGT	NED^™^	69	This study
	qASP-R	GTTTGCAGGGGGACTTTGAGT			
	ASP-P	TGGAACCTCATGCTGC			
**ASGV**	qASG-F	AGAGGACTTGCCACCAACATTT	FAM^™^	72	
	qASG-R	CACCCAAGGGCTTTTTTCAA			
	ASG-P	AGAAATGGCCCAAAGC			
**ApMV**	qApM-F	TGGTGGAGGATTACGATGAAAGTA	VIC^®^	66	
	qApM-R	TTTGAAACCCTTTCGGTCCAT			
	ApM-P	CGAAAGGTCCGAATC			

Description of forward (F) and reverse (R) primers, and TaqMan MGB (P) probes used for specific detection of *Apple stem pitting virus* (ASPV), App*le stem grooving virus* (ASGV) and *Apple mosaic virus* (ApMV) in the singleplex and multiplex RT-qPCR assays

### Development of the multiplex assay

Concentration of MgCl_2_ in the reaction mix was optimized by additional use of 2 mM and the incubation of the reverse transcription step was performed at 42°C for 30 min. Primers and probes for all viruses were used at concentrations of 200 nM and 100 nM, respectively. For both multiplex and singleplex assays 20 *μ*L reaction volumes were used and the amplification profile was 2 min at 95°C, followed by 40 cycles of 95°C for 15 s and 60°C for 1 min (One Step PrimeScript RT-PCR Kit Perfect Real Time, Takara). All singleplex and multiplex assays were performed in duplicates and at least in two individual runs, using 150 ng of RNA template on a StepOnePlus Real-Time PCR System (Applied Biosystems). All appropriate controls were included, and ROX was used as passive reference dye.

Tenfold serial dilutions of the standards were prepared in RNAse-free type 1 Milli-Q H_2_O (Merck) and mixed with 150 ng/*μ*L RNA extracted from pear and apple samples testing virus negative. Serial 10-fold dilutions of RNA extracted from a naturally triple infected plant (B71) were also prepared in virus-free RNA at a final concentration of 150 ng/*μ*L.

Competition between amplicon detection in the multiplex assay was examined by using mixtures of 10^3^ or 10^4^ copies of one target and 10^7^ copies of each of the other two targets, in the presence of 150 ng/*μ*L virus-negative tested apple or pear RNA.

Sensitivity, efficiency and linearity of the qPCR assays were estimated by constructing standard curves.

## Results

### Selection of RNA purification method

TRI-based protocols applied in pear tissues produced low quality viscous RNA. Protocols based on commercial kits resulted in ratios OD_260_/OD_280_ from 1.9 to 2.7, OD_260_/OD_230_ from 0.1 to 1.5 and yields from 18.6 to 33ng/μL (S1 Table). The CTAB method resulted in high RNA yield and ratios OD_260_/OD_280_ and OD_260_/OD_230_ 2 and 2.7, respectively, and it was adopted for all RNA extractions conducted in this study (S2 Table). Gel electrophoresis of RNAs with OD_260_/OD_280_ ratio above 1.9 showed good integrity (S2 Table).

### Multiplex RT-qPCR optimization

Testing of all reference isolates by the new singleplex and multiplex assays showed that each set of primers and probe resulted in specific amplification of the target virus with no cross reactions being observed. Moreover, no detection was recorded when non-target viroid and virus isolates as well as virus negative tested apple, pear and quince tree species were analyzed by singleplex and multiplex RT-qPCR. The addition of extra 2 mM MgCl_2_ in the multiplex reaction containing each of the three targets at high concentration (10^8^ copies), resulted in a slight reduction of the cycle thresholds (Ct), and a higher reporter fluorescence. Thus, the addition of 2 mM MgCl_2_ was adopted in the assay protocol.

### Sensitivity, efficiency and comparison of real-time multiplex to real-time singleplex and conventional RT-PCR assays

Sensitivity of RT-qPCR assay was examined by estimating the detection limit for each target using serially diluted i) standards and ii) RNA from triple infected B71 apple tree, into virus-negative tested apple and pear RNA.

When ten-fold serial dilutions of RNA standards for each virus were used into either apple or pear RNA, detection end points for multiplex assay were 10^3^ copies for ASGV, and 10^2^ copies for each of ASPV and ApMV (Table 3). Similar detection limits for all three viruses were recorded by singleplex assays applied in the same standard samples (Table 3). In contrast, detection limits of conventional RT-PCRs applied to the same RNA standards were 10^5^ copies for ApMV and ASPV, and 10^6^ copies for ASGV (Table 3 and S1 Fig). Multiplex RT-qPCR on serially diluted RNA from the naturally triple infected plant B71 in apple RNA, detected all three viral targets up to a 10^−4^ dilution. Conventional RT-PCR assays on the same dilutions detected ApMV and ASPV up to a 10^−2^ dilution, and ASGV up to a 10^−1^ dilution (Table 3 and S1 Fig).

**Table 3:** Comparison of sensitivity among multiplex and singleplex RT-qPCR and conventional RT-PCR.

Target	Multiplex RT-qPCR		Singleplex RT-qPCR		Conventional RT-PCR	
	Copy number	RNA dilution	Copy number	RNA dilution	Copy number	RNA dilution
**ASPV**	10^2^	10^−4^	10^2^	10^−4^	10^5^	10^−2^
**ApMV**	10^2^	10^−4^	10^2^	10^−4^	10^5^	10^−2^
**ASGV**	10^3^	10^−4^	10^2^	10^−4^	10^6^	10^−1^

Sensitivity of multiplex RT-qPCR and comparison to those of RT-qPCR and conventional RT-PCR assays, as estimated using tenfold serially diluted standards of *Apple stem pitting virus* (ASPV), *Apple mosaic virus* (ApMV) and *Apple stem grooving virus* (ASGV), and tenfold serially diluted total RNA extracted from a naturally triple infected tree. Serial 10-fold dilutions were prepared into RNA extracted from virus-negative tested apple tissue.

**Fig 1:**
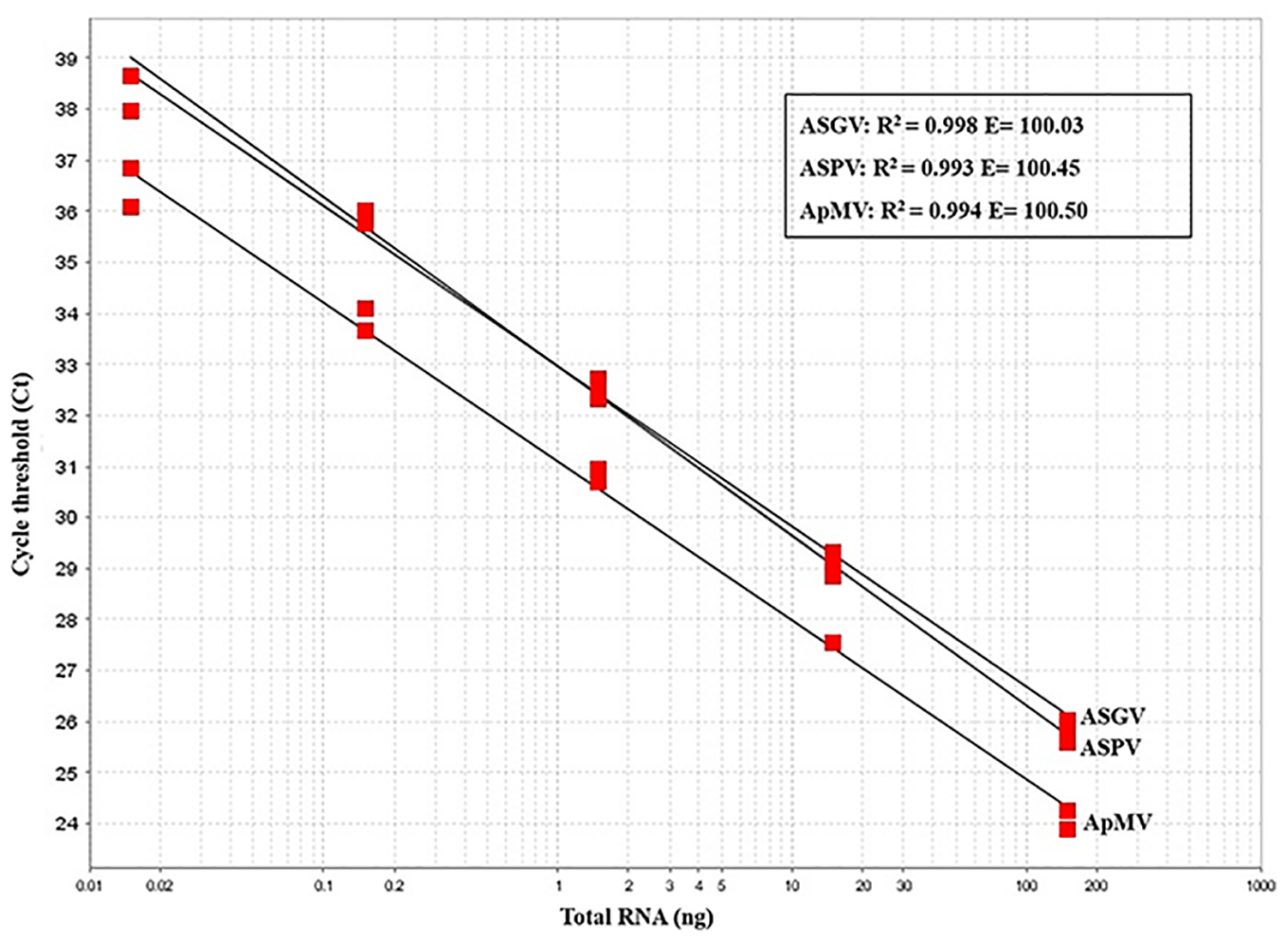
Standard curves obtained by multiplex RT-qPCR assay. Standard curves were constructed during simultaneous detection of the three viruses (ASPV, ApMV and ASGV) in serial 10-fold dilutions of the natural triple infected B71 tree. Dilutions were prepared in RNA extracted from a virus tested negative apple tree. R^2^, regression coefficient value; E, amplification efficiency.

Detection efficiencies of the multiplex assay using standards were 98.9%, 99.6% and 97.8% for ApMV, ASPV and ASGV, respectively, and regression coefficient values (R^2^) ranged from 0.98 to 1. Efficiencies and R^2^ values using RNA extracted from the naturally infected B71 tree were 100% and 0.99, respectively for all virus targets. Standard curves constructed for each target are presented in Fig 1.

### Competition among targets

**Fig 2:**
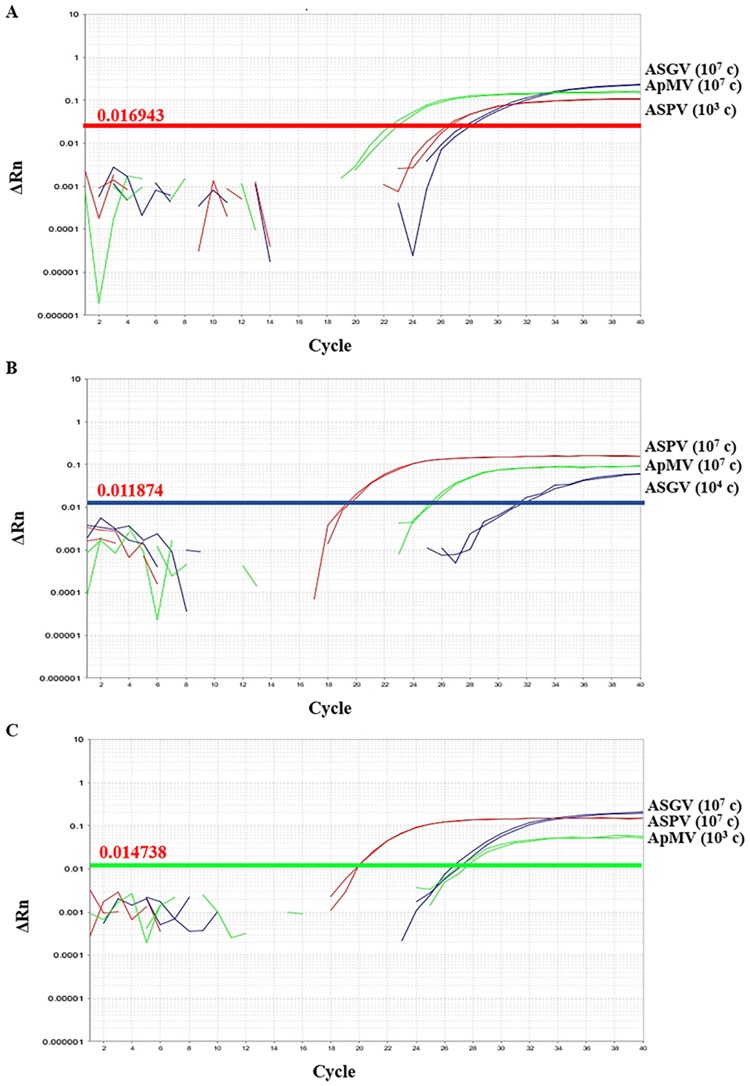
Simultaneous detection of ASPV, ApMV and ASGV by multiplex RT-qPCR in three different copy number combinations of standards. Multiplex RT-qPCR amplification plots in samples containing a high copy number of each of the two targets mixed with a relatively low copy number of the third target and diluted in RNA derived from virus negative tested apple tissue. A, ASPV at a low copy number concentration; B, ASGV at a low copy number concentration; C, ApMV at a low copy number concentration. Estimated copy numbers are denoted in parentheses. Threshold lines presented, correspond to the low copy number targets.

No apparent competition between targets for detection was recorded in the multiplex assay when 10^7^ copies of each of the two targets were mixed with the detection limit copy number of the third target into apple derived virus-tested negative RNA (Fig 2). Similar results were obtained when the mixtures were diluted into pear virus-negative tested RNA except from ASGV. For the latter, the difference in copy number between the other two targets and ASGV had to be decreased to 10^2^ copies (10^4^ copies of ASGV and 10^6^ copies of ApMV and ASPV) indicating an inhibitory or competition effect of pear tissue in ASGV amplification (S2 Fig).

### Validation of the multiplex assay

A total of 36 samples derived from 22 pear, 12 apple, one quince and one *Pyrus amygdaliformis*, were collected from commercial orchards located in different parts of Greece and tested in parallel by conventional RT-PCR and the newly developed multiplex RT-qPCR. ASPV, ASGV and ApMV were detected by RT-qPCR in 25, 18 and 11 samples, respectively (Table 4). However, 3 of the 25 samples positive for ASPV and 7 of the 18 samples positive for ASGV were negative by the conventional assays. In contrast, ASPV was detected in two pear samples from different geographical regions of Greece with conventional RT-PCR but not by multiplex or singleplex RT-qPCR. The aforementioned PCR products were sequenced and the *in silico* analysis showed four mismatches with the reverse qPCR primer. Triple infections were detected in nine samples whereas ASPV-ASGV and ASPV-ApMV double infections were detected in seven and four samples, respectively. In the same samples, seven triple, four double ASPV-ASGV and two double ASPV-ApMV infections were detected by conventional assays. No ASGV-ApMV mixed infection was detected in any of the tested trees (Table 4).

**Table 4:** Evaluation of the multiplex RT-qPCR.

s/n	Sample	Host	Geographic region	Symptoms	Multiplex RT-qPCR			Conventional RT-PCR		
					ASPV	ASGV	ApMV	ASPV	ASGV	ApMV
1	6006	Pear	Peloponnese	Fire blight symptomatology	34.99	35.02	-	-	-	-
2	G02	*P*.*amygdaliformis*	Central Greece	Symptomless	-	-	-	-	-	-
3	3492	Apple	Peloponnese	Graft necrosis	20.03	21.74	25.58	+	+	+
4	B65	Pear	Peloponnese	Reduced leaf size, short internodes	23.93	-	36.66	+	-	+
5	3096	Pear	Crete	Absence of vegetative development	-	-	-	+	-	-
6	5847	Pear	Thessaly	Reduced leaf size, chlorosis, stunting	33.96	35.35	-	+	-	-
7	KY1	Quince	Central Greece	Symptomless	-	-	-	-	-	-
8	2814	Apple	Macedonia	Chlorosis, leaf curl	22.87	23.93	27.64	+	+	+
9	MT	Apple	Peloponnese	Symptomless	21.52	23.93	26.51	+	+	+
10	Tragic	Pear	Central Greece	Stunting	33.7	-	-	+	-	-
11	M4	Apple	Central Greece	Symptomless	23.41	23.63	27.17	+	+	+
12	EVA2	Apple	Central Greece	Symptomless	21.84	-	-	+	-	-
13	2970	Pear	Thessaly	Necrosis of branches	33.14	35.44	-	+	-	-
14	ELEA2	Apple	Central Greece	Symptomless	23.05	-	36.04	+	-	+
15	WIL	Pear	Central Greece	Symptomless	-	-	-	-	-	-
16	4176	Pear	Central Greece	Symptomless	29.83	-	-	+	-	-
17	EP913	Pear	Epirus	Wilting	32.35	32.65	29.38	+	-	+
18	B39	Apple	Thessaly	Cankered branches	20.56	23.71	27.34	+	+	+
19	4215	Pear	Macedonia	Necrotic spots, stunting	30.85	33.55	-	+	-	-
20	EVP2	Pear	Central Greece	Symptomless	-	-	-	+	-	-
21	3248	Pear	Central Greece	Leaf spots	36.63	-	-	-	-	-
22	B47	Pear	Macedonia	Cankered limbs, shoot necrosis	36.06	-	-	-	-	-
23	PEL5	Apple	Thessaly	Reduced fruit size	24.65	24.04	20.88	+	+	+
24	KPY	Pear	Central Greece	Symptomless	-	-	-	-	-	-
25	3363	Pear	Thrace	Reduced leaf and branch size	35.82	36.53	-	-	-	-
26	4608	Pear	Thessaly	Reduced fruit size	-	-	-	-	-	-
27	APL5	Apple	Macedonia	Symptomless	34.67	36.02	35.81	-	-	+
28	15643	Pear	Macedonia	Limbs with cankers	-	-	-	-	-	-
29	B70	Apple	Macedonia	Mosaic	25.89	24.05	24.33	+	+	+
30	M3	Apple	Epirus	Symptomless	-	-	-	-	-	-
31	M1	Apple	Central Greece	Symptomless	24.33	25.87	-	+	+	-
32	M2	Apple	Central Greece	Symptomless	22.15	24.99	-	+	+	-
33	A1	Pear	Central Greece	Symptomless	-	-	-	-	-	-
34	A2	Pear	Central Greece	Symptomless	26.63	25.95	-	+	+	-
35	A3	Pear	Central Greece	Symptomless	-	-	-	-	-	-
36	A4	Pear	Central Greece	Symptomless	25.3	26.28	-	+	+	-
**Total**^a^					25	18	11	22	11	11

Total RNAs isolated from thirty-six samples of pome trees derived from commercial orchards, were analysed by the developed multiplex RT-qPCR and conventional RT-PCR assays. Comparison between methods was performed on the same RNA. Samples detected positive are indicated by cycle thresholds (Ct) and (+), for multiplex RT-qPCR and conventional RT-PCR, respectively.

^a^ Number of samples in which at least one virus was detected

**Fig 3:**
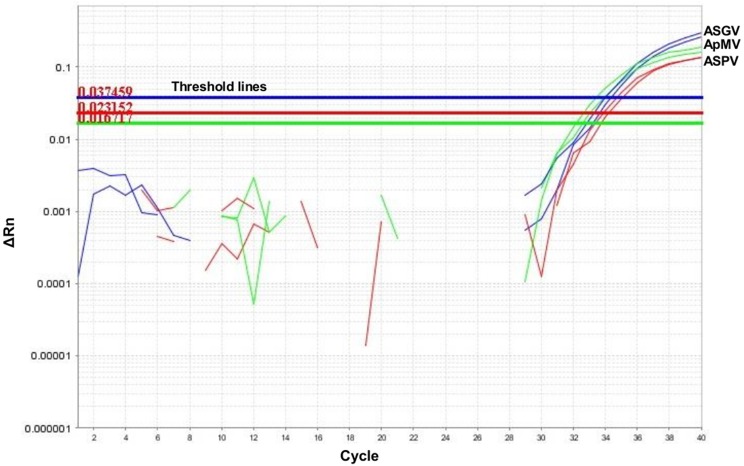
Simultaneous detection of the three viruses during simulation of composite sample examination. Tissue derived from the triple infected B71 tree was mixed into pear tissue tested virus negative at a ratio of 1:150 before RNA isolation.

Moreover, simulation of composite sample examination resulted in detection of all three targets by multiplex RT-qPCR in all ratios from 1:25 to 1:150 (Fig 3).

Reproducibility of the method was evaluated through calculation of mean Ct values and standard deviations that were obtained by analysis of serial dilutions of the triple infected B71 RNA in three biological replicates, and of serial dilutions of RNA transcripts in two individual runs (Table 5). *C*_t_ standard deviation range is presented in Table 5.

**Table 5:** Reproducibility of simultaneous detection of ApMV, ASPV and ASGV with multiplex RT-qPCR.

Samples		Multiplex real-time PCR mean *C*_t_ values		
		ApMV	ASPV	ASGV
Three biological replicates of triple infected B71 RNA dilutions	B71	25.07 (0.29)	25.30 (0.66)	25.23 (0.72)
	10^−1^	28.82 (0.53)	27.77 (0.15)	29.10 (0.19)
	10^−2^	31.05 (0.26)	30.78 (0.09)	32.84 (0.36)
	10^−3^	34.26 (0.36)	33.20 (0.38)	37.17 (0.31)
	10^−4^	37.07 (0.12)	36.62 (0.28)	Na
Two individual runs of serial dilutions of RNA transcripts	10^6^ copies	25.03 (0.4)	24.07 (0.12)	31.4 (0.71)
	10^5^ copies	28.40 (0.49)	27.41 (0.34)	34.34 (0.19)
	10^4^ copies	30.74 (0.35)	29.81 (0.73)	35.58 (0.06)
	10^3^ copies	32.58 (0.49)	32.04 (0.74)	37.23 (0.49)
	10^2^ copies	36.67 (0.85)	35.88 (0.92)	Na

Reproducibility of the method as evaluated through calculation of mean C_t_ values and standard deviations (in brackets) of serial dilutions of the triple infected B71 RNA in three biological replicates and of RNA transcript serial dilutions in two individual runs. Serial 10-fold dilutions were prepared into RNA extracted from virus-negative tested pear tissue.

Na: not applicable

## Discussion

Despite of the high number of publications dealing with the development of multiplex quantitative PCR protocols for the detection of plant viruses and viroids, protocols for the simultaneous detection of tree pathogens are scarce [39–42]. In principal, the simultaneous detection of different pathogens in one reaction combines the increased sensitivity and speed of qPCR with the reduced labour time and cost of multiplexing, increasing the number of samples processed in a certain amount of time [27]. However, the optimization of multiplex assays is more demanding compared to singleplex tests, and more advanced and expensive laboratory equipment is required. Moreover, above of a certain number of targets there is often a drop of the qualitative characteristics of the assay like sensitivity and efficiency [39, 40, 42].

Woody species tissues are rich in polysaccharides and polyphenols resulting in RNA extracts containing high amounts of PCR inhibitors. The CTAB extraction protocol [37] which is well known for its ability to extract nucleic acids from a wide range of polysaccharide- and polyphenol-rich woody tissues [39–41] performed in all cases better than the other three commercial protocols tested in terms of both RNA yield and quality.

High genetic variability within a virus species constitutes a challenge for the design of probes able to detect the whole range of variants. The retrieval of ApMV, ASPV and ASGV complete and partial genome sequences from GenBank, revealed nucleotide sequence divergence up to 88%, within each virus, respectively. Thus, specific TaqMan probes incorporating MGB quenchers were designed targeting the most conserved regions of each virus. MGB conjugated DNA probes form duplexes of increased stability with single-stranded DNA targets, thus shorter probes can be designed to hybridize to all known isolates [43]. All probes detected the four reference ApMV, ASPV and ASGV isolates tested. However, validation of the assay revealed two ASPV isolates that were detected by the conventional but not by the multiplex assay developed here. Direct nucleotide sequence characterization of the two respective ASPV RT-PCR products revealed four mismatches with the reverse qPCR primer. Moreover, phylogenetic analysis showed that the two isolates belonged to a newly characterized ASPV group and are organized along with other ASPV isolates from China.

Limits of detection were calculated by two ways: i) serial dilutions of each standard in RNAs derived from tissues tested virus negative and ii) serial dilutions of RNAs extracted from tissue derived from a naturally triple infected plant. Amplification efficiencies of 100% and regression coefficient values (R^2^) higher than 0.99 were obtained during simultaneous three target detection in a natural triple virus infection, demonstrating accuracy and linear response of the assay over a wide range of dilutions and performance that was not affected by competition among reaction components. These characteristics further demonstrated that the developed assay additionally to detection constitutes a reliable quantitative tool suitable for population or competition studies among the three viruses in mixed infections in a variety of pome tree hosts. Moreover, the low standard deviation of the mean *C*_t_ which was recorded in most cases proved the reproducibility of the method (Table 5).

Comparative analysis was also performed in serial dilutions of RNA extracted from natural triple infected tissue and standards-RNA, into virus tested negative RNAs and showed that the multiplex assay was in both cases at least 100 times more sensitive than conventional RT-PCR protocols for virus detection. Moreover, the robustness and reliability of the method were further verified by the low degree of competition among targets, as tested using all mixture combinations of two standards in high copy numbers and one standard in a copy number near the limit of detection of the assay; the three targets were simultaneously detected in all cases, in the presence of RNAs from tissue tested virus negative.

Moreover, the multiplex assay confirmed its suitability for composite sample examination, which is a common practice in routine analysis during certification programs, as it was able to detect all targets simultaneously even when tissue bearing triple infection was mixed with tissue tested virus negative at a ratio of 1:150.

The usefulness of the multiplex RT-qPCR assay was further demonstrated during evaluation, where the majority of the detected infections contained mixes of three or two viruses. Furthermore, ASPV and ASGV were detected in more field samples (two and seven, respectively) than those detected by the conventional protocols (Table 4).

In conclusion, a CTAB extraction protocol was combined with a multiplex single-tube RT-qPCR assay using TaqMan MGB probes, for the simultaneous detection of three important pome fruit viruses. Further experiments, in the form of ring tests including a larger range of reference virus isolates and pome tree species and cultivars, are required before the assay presented here could be recommended as a reference detection method. However, the multiplex RT-qPCR displayed high sensitivity, efficiency and improved reliability and has the capacity to assist large-scale surveys for disease management and certification programs. To the best of our knowledge this is the first report of a single-tube RT-qPCR assay for the simultaneous detection of three pome fruit tree viruses.

## Supporting information

S1 Fig Comparison of sensitivity among multiplex RT-qPCR and conventional RT-PCR. Amplification plots of multiplex RT-qPCR (panels A, C) and product electrophoresis gels of conventional RT-PCR assays, (panels B, D) derived from assays applied on tenfold serially diluted standards (panels A, B) of *Apple stem pitting virus* (ASPV), *Apple mosaic virus* (ApMV) and *Apple stem grooving virus* (ASGV), and tenfold serially diluted total RNA extracted from a naturally triple infected plant (panels C, D). Serial 10-fold dilutions were prepared into RNA extracted from virus-negative tested apple tissue. L: 1 Kb Plus DNA Ladder (Invitrogen).
(TIF)

S2 Fig Simultaneous detection of ApMV, ASPV and ASGV by multiplex RT-qPCR in mixture containing 10^4^ copies of ASGV and 10^6^ copies of each of ApMV and ASPV. The mixture was diluted into pear virus-negative tested RNA. Threshold line presented, corresponds to ASGV.
(TIF)

S1 Table Reference isolates. Description of *Apple mosaic virus* (ApMV), *Apple stem grooving virus* (ASGV) and *Apple stem pitting virus* (ASPV) reference isolates used for the multiplex RT-qPCR assay development.
(DOCX)

S2 Table Comparison of RNA extraction protocols. Evaluation of the extraction protocols tested in terms of yield, purity (ratios OD_260_/OD_280_ and OD_260_/OD_230_) and integrity of the isolated RNA after measurements in a nanophotometer and gel electrophoresis. RNA was isolated from 70 mg aliquots of the same tissue homogenate derived from leaf disks of a pear tree.
(DOCX)
