# Supplementary figures and images for: Simultaneous detection of three pome fruit tree viruses by one-step multiplex quantitative RT-PCR
Source: PLoS One. 2017 Jul 27;12(7):e0180877. doi: 10.1371/journal.pone.0180877 (PMC5547701; doi:10.1371/journal.pone.0180877)

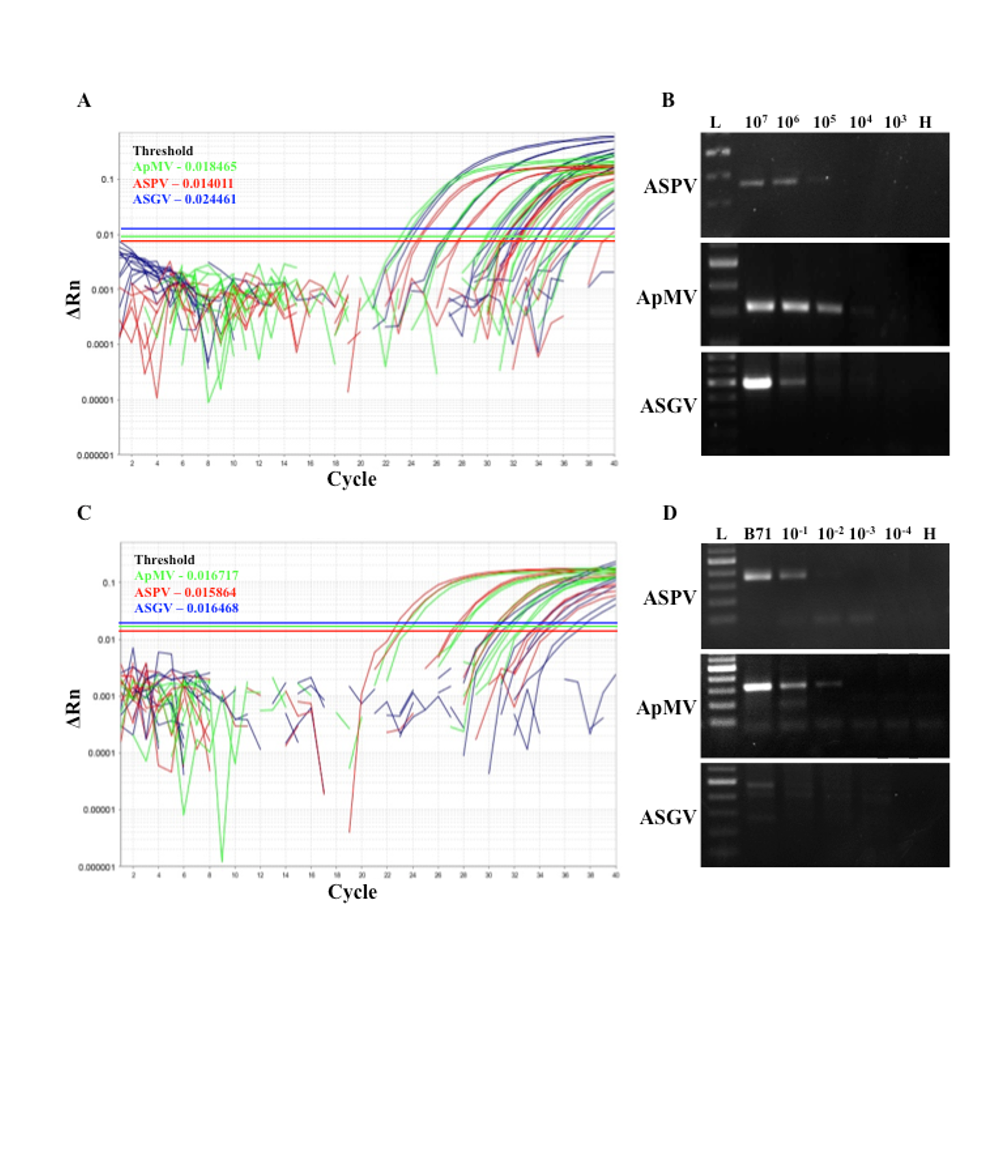

Supplement: S1 Fig — Amplification plots of multiplex RT-qPCR (panels A, C) and product electrophoresis gels of conventional RT-PCR assays, (panels B, D) derived from assays applied on tenfold serially diluted standards (panels A, B) of Apple stem pitting virus (ASPV), Apple mosaic virus (ApMV) and Apple stem grooving virus (ASGV), and tenfold serially diluted total RNA extracted from a naturally triple infected plant (panels C, D). Serial 10-fold dilutions were prepared into RNA extracted from virus-negative tested apple tissue. L: 1 Kb Plus DNA Ladder (Invitrogen). (TIF) [file pone.0180877.s001.tif]

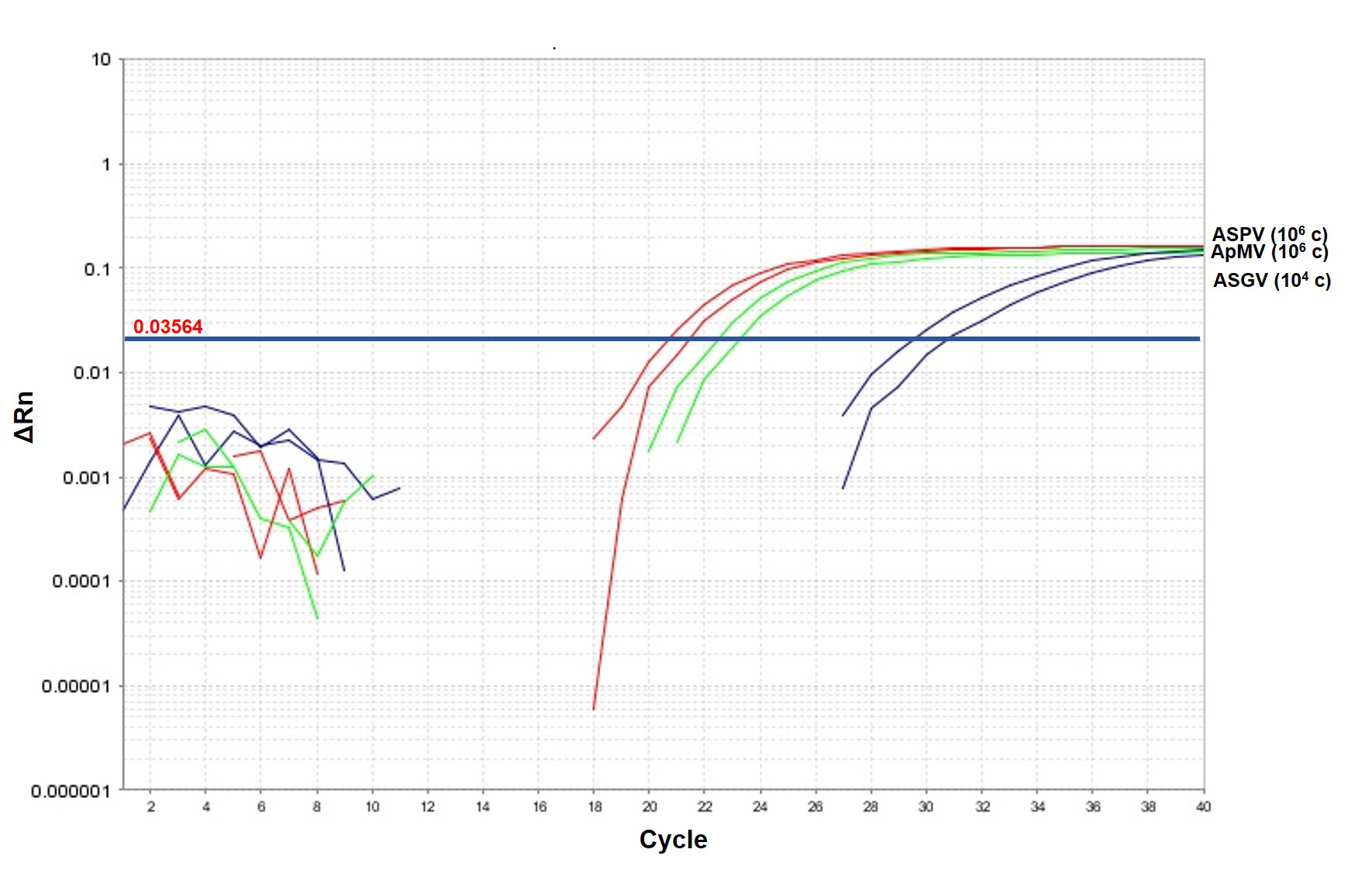

Supplement: S2 Fig — The mixture was diluted into pear virus-negative tested RNA. Threshold line presented, corresponds to ASGV. (TIF) [file pone.0180877.s002.tif]
