# Supplementary material for: Simultaneous detection of three pome fruit tree viruses by one-step multiplex quantitative RT-PCR
Source: PLoS One. 2017 Jul 27;12(7):e0180877. doi: 10.1371/journal.pone.0180877 (PMC5547701; doi:10.1371/journal.pone.0180877)
Supplement: S1 Table — Description of Apple mosaic virus (ApMV), Apple stem grooving virus (ASGV) and Apple stem pitting virus (ASPV) reference isolates used for the multiplex RT-qPCR assay development. (DOCX) [file pone.0180877.s003.docx]

**S1 Table. Reference isolates**

| **Pathogen** | **Sample ID** | **Original ID** | **Origin** | **Source/Reference** |
| --- | --- | --- | --- | --- |
| **ApMV** | ApMV7 | P1LA12 | France | CTIFL |
| **ASPV** | ASPV9 | LX156 K1Ca | France | CTIFL |
|  | ASPV10 | LX86 | France | CTIFL |
|  | ASPV12 | PO13357 | France | CTIFL |
| **ASGV** | ASGV13 | X680 B3a | France | CTIFL |
|  | ASGV14 | X5119 H3a | France | CTIFL |
| **ApMV, ASPV, ASGV in natural triple infection** | B71 | B71 | Greece | Benaki Phytopathological Institute |

Description of *Apple mosaic virus* (ApMV), *Apple stem grooving virus* (ASGV) and *Apple* *stem* *pitting* *virus* (ASPV) reference isolates used for the multiplex RT-qPCR assay development
