# Supplementary material for: Simultaneous detection of three pome fruit tree viruses by one-step multiplex quantitative RT-PCR
Source: PLoS One. 2017 Jul 27;12(7):e0180877. doi: 10.1371/journal.pone.0180877 (PMC5547701; doi:10.1371/journal.pone.0180877)
Supplement: S2 Table — Evaluation of the extraction protocols tested in terms of yield, purity (ratios OD260/OD280 and OD260/OD230) and integrity of the isolated RNA after measurements in a nanophotometer and gel electrophoresis. RNA was isolated from 70 mg aliquots of the same tissue homogenate derived from leaf disks of a pear tree. (DOCX) [file pone.0180877.s004.docx]

**S2 Table:** **Comparison of RNA extraction protocols**

| RNA extraction protocol | Yield (ng/μL) | OD_260_/OD_280_ | OD_260_/OD_230_ | Integrity  (gel visualisation) |
| --- | --- | --- | --- | --- |
| TRI Reagent^®^ Solution (Ambion^TM^) | 100 | 1.3 | 0.1 | NA |
| TRI modified  by Rowhani and co-workers | <2 | NA | NA | NA |
| Purelink^®^ RNA Mini Kit (Ambion^TM^) | 33 | 1.9 | 0.5 | good |
| Purelink^®^ modified by  MacKenzie and co-workers | 66 | 2.2 | 0.1 | good |
| Purelink^®^ modified by  López-Fabuel and co-workers | 18.6 | 2.7 | 1.5 | good |
| CTAB-modified by  Gambino and co-workers | 300 | 2.0 | 2.5 | good |

**NA**: Not applicable

Evaluation of the extraction protocols tested in terms of yield, purity (ratios OD_260_/OD_280_ and OD_260_/OD_230_) and integrity of the isolated

RNA after measurements in a nanophotometer and gel electrophoresis. RNA was isolated from 70 mg aliquots of the same tissue

homogenate derived from leaf disks of a pear tree.
